# Supplementary material for: Association of Korean Healthy Eating Index and Sleep Duration with Obesity in Korean Adults: Based on the 7th Korea National Health and Nutrition Examination Survey 2016–2018
Source: Nutrients. 2024 Mar 14;16(6):835. doi: 10.3390/nu16060835 (PMC10975321; doi:10.3390/nu16060835)
Supplement: Supplementary file 1 [file nutrients-16-00835-s001.zip › nutrients-2884974-supplementary.pdf]

Table S1. Korean Healthy Eating Index components and lifecycle-specific standard for scoring.

Components	Score range	Standard for maximum score	Standard for minimum score
**Adequacy (8)**			
Have breakfast*	0-10	5-7 times/w	0 times/wk
Mixed grains intake*, †	0-5	≥ 0.3 serving/d	0 serving/day
Total fruits intake†	0-5	Men aged 19-64 years: ≥ 3serving/d<br>Men aged 65 years and overs: ≥2serving/d<br>Women aged 19-64 years: ≥ 2 serving/d<br>Women aged 65 years and overs: ≥ 1 serving/d	0 serving/day
Fresh fruits intake*, †	0-5	Men aged 19-64 years: ≥ 1.5 serving/d<br>Women aged 19-64 years: ≥ 1 serving/d<br>Men aged 65 years and overs: ≥ 1 serving/d<br>Women aged 65 years and over: ≥ 0.5 serving/d	0 serving/day
Total vegetables intake†	0-5	Men and women aged 19-64 years: ≥ 8 serving/d<br>Men aged 65 years and overs: ≥ 8 serving/d<br>Women aged 65 years and overs: ≥ 6 serving/d	0 serving/day
Vegetables intake excluding kimchi and pickled vegetables intake*, †	0-5	Men and women aged 19-64 years: ≥ 5 serving/d<br>Men aged 65 years and overs: ≥ 5 serving/d<br>Women aged 65 years and overs: ≥ 3 serving/d	0 serving/day
Meat, fish, eggs and beans intake†	0-10	Men aged 19-64 years: ≥ 5 serving/d<br>Women aged 19-64 years: ≥ 4 serving/d<br>Men aged 65 years and overs: ≥ 4 serving/d<br>Women aged 65 years and overs: ≥ 2.5 serving/d	0 serving/day
Milk and milk products intake†	0-10	≥ 1 serving/d	0 serving/day
**Moderation (3)**			
Percentage of energy from saturated fatty acid†, ‡	0-10	≤ 7% of total energy intake	> 10% of total energy intake
Sodium intake†, §	0-10	≤ 2000 mg/d	> 6500 mg/day
Percentage of energy from sweets and beverages†	0-10	≤ 10% of total energy intake	> 20% of total energy intake
**Energy balance (3)**			
Percentage of energy from carbohydrate†, §	0-5	55-65% of total energy intake	< 50% or > 75% of total energy intake
Percentage of energy intake from fat†, ‡, §	0-5	15-30% of total energy intake	< 10% or > 35% of total energy intake
Energy intake†, §	0-5	75-125% of the estimated energy intake requirement (EER)	< 60% or > 140% of EER

EER, estimated energy requirement; WHO, World Health Organization; FAO, Food and Agriculture Organization.
*Dietary guidelines for Korean adults; †Dietary Reference Intake for Koreans 2015; ‡Recommendation criteria of WHO/FAO; §15 or 85 percentile value in Korean adults aged 19 years and over.
